# Medical education leadership: from diversity to inclusivity

**DOI:** 10.3205/zma001311

**Published:** 2020-03-16

**Authors:** Constance LeBlanc, Lyn K. Sonnenberg, Sharla King, Jamiu Busari

**Affiliations:** 1 Dalhousie University, Department of Emergency Medicine and Associate, Halifax, NS, Canada; 2 Dalhousie University, Associate Dean of Continuing Professional Development and Medical Education Research, Halifax, NS, Canada; 3 University of Alberta, Department of Pediatrics, Edmonton, AB, Canada; 4 University of Alberta, Associate Dean of Educational Innovation & Academic Technologies, Edmonton, AB, Canada; 5 Glenrose Rehabilitation Hospital, Edmonton, AB, Canada; 6 University of Alberta, Faculty of Education, Department of Educational Psychology, Faculty of Education, Edmonton, AB, Canada; 7 University of Alberta, Direktor of Health Sciences Education and Research Commons, Edmonton, AB, Canada; 8 University of Maastricht, Faculty of Health, Medicine and Life Sciences, Department of Educational Development and Research, Maastricht, the Netherlands; 9 Zuyderland Medical Center, Department of Pediatrics, Heerlen, the Netherlands

**Keywords:** leadership, diversity, inclusiveness, medical education

## Abstract

Both in Canada and globally, medical schools are prioritizing diversity in medical education. The ensuing development of innovative approaches to augmenting the representation, comfort, and success of students from under-represented groups has been increasing. Curricula have also expanded to better prepare graduates for the realities of effectively meeting the needs of a diverse patient population. Leadership has however, not kept up with this progress. Evidence shows that diverse leadership teams develop innovative solutions to complex problems, recruit and retain the best talent, and remain relevant to the communities they serve.

Our international conference workshop included a literature review on the current state of diversity in medical education and in leadership for medical educators, and case-based models of lived experiences to initiate conversations in three different facets of diversity to stimulate reflection, engagement and discussion. The oft-forgotten side of the conversation in conference offerings, the audience’s perspective, was purposefully included in planning the workshop and presenters adhered to this principle throughout the session.

Participants recognized the importance of addressing diversity with leadership in medical education. Themes included the need for communication training, cultural education, sharing these data more broadly with faculty in medical education and continuing these conversations. A final theme “we will never represent all minorities”, led us to a conclusion that a culture of inclusivity and not diversity would be required to successfully meet this challenge.

## Zusammenfassung

Diversität in medizinischer Ausbildung wird zunehmend zum Schwerpunkthema an medizinischen Hochschulen, sowohl in Kanada als auch weltweit. Teil dieser Entwicklung sind innovative Ansätze, die das Ziel haben, die Anzahl, den Komfort und letztendlich den Erfolg von Studenten aus Minderheiten zu verbessern. Auch Lehrpläne werden diesem Schwerpunkt angepasst, damit zukünftige Fachkräfte im Gesundheitswesen effektiver den Bedürfnissen einer diversen Patientenpopulation gerecht werden können. Medizinische Führung indessen hinkt diesem Fortschritt hinterher. Wissenschaftliche Forschung belegt, dass leitende Teams mit diversen Mitgliedern innovative Lösungen für komplexe Probleme finden, die besten Arbeitskräfte anstellen und beibehalten und über lange Zeiträume zutreffende Fürsorge für ihre entsprechende Gemeinschaft anbieten.

In unserem internationalen Konferenz-Workshop haben wir uns einen Literaturüberblick über den derzeitigen Stand von Diversität in medizinischer Ausbildung und im Führungswesen der medizinischen Schulung verschafft. Zusätzlich dazu haben wir Musterfälle auf reellen Fallbeispielen basierend erstellt, die den Zweck hatten, Dialoge zu drei Facetten der Diversität einzuleiten. Die Intention hinter solchen Dialogen war es, zu Reflektion, Engagement und Diskussionen anzuregen. Wir haben auch die häufig vergessene Perspektive der Zuhörer gezielt im Design unseres Workshops inkludiert. Die Präsentatoren haben diesen Aspekt dementsprechend während des gesamten Workshops beachtet.

Teilnehmer haben die Relevanz der Diversität im Führungspersonal medizinischer Ausbildner anerkannt. Die Hauptthemen waren: die Notwendigkeit von Kommunikationstraining und Schulung zu unterschiedlichen Kulturen, im Weiteren das weiträumige Teilen von diesen Daten für Pädagogen in Medizin und auch die Fortsetzung von Dialogen. Ein weiteres Thema bezüglich „man kann niemals alle Minderheiten repräsentieren“, hat uns zu der Schlussfolgerung geführt, dass es eine Betriebskultur von Inklusivität und nicht Diversität benötigt, um dieses Thema erfolgreich zu bearbeiten.

## Introduction

Medical programs in several countries, including Canada, the Netherlands, United Kingdom and the United States of America, are now prioritizing diversity in medical education. In western societies, the practice of Medicine has traditionally been by white, male, heterosexual and affluent practitioners, however in a world that has changed politically, economically, and technologically, the landscape of healthcare delivery has also undergone transformation [1]. Nowadays, training to be a physician is not just for the elites in society but is open to everyone based on academic merit, provided they meet criteria for admission into medical school. Furthermore, the science of medicine is not limited to the expertise of the physician anymore; neither is the access to medical information and treatments confined to libraries and hospitals, as technology and social media have disrupted the hegemony of the medical profession. Political upheavals, mass migration, and human displacements, due to natural and man-made disasters, have led to increased diversity in the composition of populations, their health care needs, and demands on health resources. Consequently, the education of healthcare professionals, and the management of health systems, need to accommodate these new changes to help the populations they are serving. It also means that in order to serve all patients, healthcare workforces should reflect their communities’ diversity.

### Definitions

Broadly defined, diversity is “being composed of different elements” [https://www.merriam-webster.com/dictionary/hacker]. In the contexts of this article, and our workshop described below, diversity includes sex, race, culture, religion, disability, age, and the differing abilities of teams involved as learners, planners, or leaders in health education curricula. We also endorse diversity as a management strategy and diversity as an ethical principle with respect to the equal treatment and equal opportunity for all members of society. Inclusivity can best be defined as “the practice or policy of including people who might otherwise be excluded or marginalized” (Oxford, 2019). We include in our definition of leadership all five facets of the LEADS framework [2]: Lead self, Engage others, Achieve results, Develop coalitions, Systems transformation. Leadership in health, and leadership in inclusivity, are relevant through each of these lenses.

In response to population diversity and the need for inclusivity, the medical curriculum has expanded to better prepare graduates for effectively meeting the needs of a diverse patient population. At the same time, the ensuing development of innovative approaches to augment the representation, comfort, and success of students from under-represented groups has been steadily increasing [3]. Attention is required to ensure diversity in case studies, discussions, communication skills training, simulations, and in extracurricular activities. Moreover, in higher education, diversity has also been applied to research. However, in contrast to other professional domains within medicine, leadership has not kept up with the alacrity of this progress. Evidence reveals that diversity in leadership teams facilitates innovative solutions to complex problems, helps recruit and retain best talent, and remain relevant to the communities they serve [4]. Although necessary for success, diversity within healthcare leadership education remains a step behind creating leadership education to meet these needs. 

The Toronto International Summit on Leadership Education for Physicians (TISLEP) is the educational flagship of sanokondu, an international community of practice dedicated to the creation and dissemination of open educational resources to support leadership educators. Sanokondu aims to foster health professional leadership education worldwide collaboratively, through the development of generic competency-based leadership curriculum and activities, which can be adapted to local and regional contexts [5]. In 2018, this group included a workshop on Leading for Diversity in their October conference in Canada that we were able to facilitate [http://tislep.pgme.utoronto.ca/about/]. The annual TISLEP conference for 2019 will be entirely focused on Diversity and Inclusivity. We will provide an invited workshop at this event to further develop the concepts from our previous workshop.

## Our group

Although no group can represent all facets of diversity, ours included diversity of race, culture, age, abledness, and sex. Each member had prior interest and experience in diversity and medical education, which equipped us to plan and deliver this effective workshop. Our experiences spanned the continuum of medical education from undergraduate to postgraduate training to continuing medical education, included broad geographic representation, and was both intra-professional and interdisciplinary, providing a broader lens on our collective view of diversity in leadership. 

## Event

**Figure 1:**
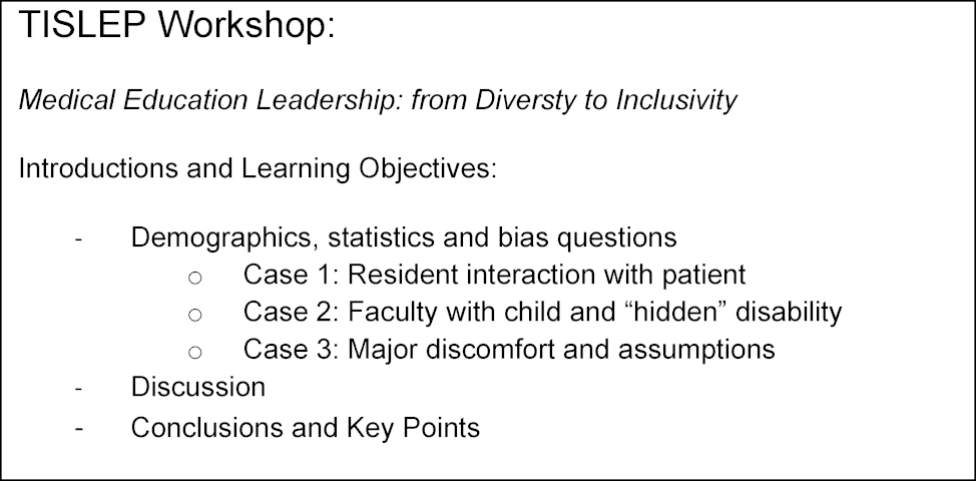
Leadership workshop in Diversity (TISLEP 2018)

To prepare for this international workshop on Diversity in Leadership for Medical Education, we met virtually to discuss our perspectives as an international and diverse group of presenters. A literature review was conducted by each of the presenters using and pertinent articles were shared and annotated for the group. Themes based on individual, lived-experiences in education, or in experiences with healthcare, were brought forth by group members and a content flow for the session was proposed. After several meetings, the outline for our workshop was set and adjuncts (slides in this case) were made to bring a cohesive feel and flow to the workshop. Our design specifically included time for dialogue with participants after each case presentation. This is a welcomed standard in Canada for all educational events provided for physicians. An overall outline of the workshop format is provided in figures 1 (Fig. 1) below.

After introduction of all group members, we presented our literature review and statistics on the current state of diversity in medical education and in leadership. Included were several questions for participants including an “Envision a Leader” ice-breaker exercise to set the stage for the lived cases. Participants were asked to picture in their minds: a president, a Chief Executive Officer, or a Dean of a Medical School. After a minute or two, participants were asked if any were female, racially or culturally diverse, or disabled. None were. This highlighted the magnitude of change required to address not only the status quo, but also our inherent biases. The introduction was presented collaboratively by the group. We then identified key ideas and outcomes of inclusiveness in serving a population and in increasing the performance of teams to set the stage for a series of conversations. 

In the three subsequent segments of the workshop each group member presented a case-based model of a lived experience to initiate conversations in three different facets of diversity to stimulate reflection, engagement, and discussion. The oft-forgotten side of the conversation in conference offerings, the audience’s perspective, was purposefully included in planning the workshop and presenters adhered to this principle throughout the session. The case-studies models included: missed opportunities for everyday leadership in inclusiveness and the impact of intersectionality on learners; tokenism vs. awareness; the hidden face of disability; the misconception of diversity, and the proposal to re-focus on inclusivity.

- The first case reported a resident who was subject to racial abuse from a patient in the Emergency Department. The presenter provided the scenario and asked for suggestions as to how best to manage the abuse. After participant contributions were exhausted, the rest of the story was shared, and the dénouement reviewed;
- Case two was a personal story of a child with a disability and perspectives of others on the situation that were not felt to portray the situation accurately due to biases and assumptions, and how many in medicine carry “hidden” disabilities. Suggestions from participants were sought and several options for mitigating these biases and for managing similar situations were proactively shared;
- The third case presented took place at a leadership meeting, where despite evident racial difference, a leader’s opinion was not sought by other high-level leaders when racial biases were discussed. Leaders at this meeting discussed strategies to mitigate racial profiling; however, discomfort with racial diversity at this meeting was so pronounced that nobody asked for the perspective of the one racially diverse leader at the table, leaving “an elephant” in the room. Approaches to opening conversations on diversity and paths to inclusivity were proposed by participants in our workshop. The end of this story was also shared with the participants.

Numerous examples of challenging situations were provided by participants and discussed in an open forum. Overall approaches to mitigating biases, to remaining culturally sensitive and moving towards inclusivity, were provided by our group and participants in equal parts. Workshop participants eagerly shared their experiences with the group, integrating their stories and anecdotes with those presented, resulting in a rich case-based discussion. 

In closing the workshop, our group provided a few key take home messages both from our cases, our experiences and those provided by workshop participants. We learned from our peers, and they from us, and the resultant sharing of ideas and approach was felt to be of value by all. Essential tools for this workshop included:

1. faculty members with an interest,
2. a welcoming venue, and
3. real cases that illustrated diversity challenges through varied lenses with
4. ample time for discussion both with and among participants, made for an effective workshop event.

## Outcomes

Quiet, yet attentive early on, participants responded enthusiastically to the challenging cases provided, and eagerly engaged in the dialogue. Many shared intimate anecdotes and stories from their professional experiences and, in so doing, revealed their discomfort, perceived lack of exposure or training, and challenges in leading for diversity. We believe that this openness stemmed from the power of vulnerability that was modeled early on in the workshop, with presenters embracing the emotions that we experienced during times of uncertainty, risk, and emotional exposure [6]. By the end of the workshop, all recognized the importance of meeting needs more broadly in medical education, with many advocating for enhanced training in this area. Themes emerged including the need for

1. communication training,
2. cultural education,
3. sharing of these data more broadly with faculty in medical education, and
4. continuing these conversations.

A final theme “we will never represent all minorities”, led to our conclusion that a culture of inclusivity, and not diversity, would be required to successfully meet this challenge [https://en.oxforddictionaries.com/definition/inclusivity].

Workshop ratings and feedback were very positive and noted, in particular, lots of new ideas shared, powerful stories, and an interesting workshop design.

## Conclusions

The process of planning and providing this event allowed us to broaden our personal perspectives on diversity. Participants were eager to share experiences and the conversational format was preferred to denser content delivery-based models. Inclusivity should be used as a term to describe our direction, as appearance and demeanor can be deceptive. 

We have initiated further dissemination of this work through local and regional workshops, national presentations, scholarly work, and programme development to meet this often-unperceived need among our leaders. 

## Competing interests

The authors declare that they have no competing interests.
